# Immune correlates of outcomes after nucleos(t)ide analogue withdrawal in chronic hepatitis B: direct evidence, mechanistic context, and candidate biomarkers

**DOI:** 10.3389/fimmu.2026.1949176

**Published:** 2026-08-25

**Authors:** Zihao Xu, Yifan Liu, Liangbin Cheng, Jun Xu

**Affiliations:** 1 School of Chinese Medicine, Hubei University of Chinese Medicine, Wuhan, Hubei, China; 2 Department of Hepatology, Hubei Provincial Hospital of Traditional Chinese Medicine Affiliated to Hubei University of Chinese Medicine, Wuhan, Hubei, China; 3 School of Basic Medical Sciences, Hubei University of Chinese Medicine, Wuhan, Hubei, China

**Keywords:** B cells, biomarkers, chronic hepatitis B, cytokines, HBsAg loss, immune complexes, nucleos(t)ide analogue withdrawal, T cells

## Abstract

Nucleos(t)ide analogue (NA) discontinuation in chronic hepatitis B (CHB) can produce two clinically divergent trajectories: a minority of patients achieve sustained hepatitis B surface antigen (HBsAg) loss or off-treatment viral control, whereas others develop hepatitis flares without virological benefit. Recent longitudinal studies have begun to define the host immune correlates of these outcomes. This Mini Review separates direct evidence from NA-withdrawal cohorts from mechanistic evidence obtained in other CHB settings. Direct human data suggest that beneficial outcomes are associated with recovery of HBsAg-specific memory B-cell activity, plasmablast expansion, stronger T follicular helper collaboration, and preserved hepatitis B virus (HBV)-specific T-cell function. In contrast, high inhibitory-receptor expression on global T cells, persistent inflammatory chemokine signatures, and greater post-flare alanine aminotransferase variability are associated with non-beneficial flares. immune complexes formed by HBsAg and antibodies to HBsAg provide an additional humoral readout that may reflect both flare risk and the quality of HBsAg decline. These observations are promising but remain exploratory: cohorts are small, flare definitions are heterogeneous, assays are not standardized, and most signatures lack external validation. Immune markers should therefore be viewed as candidate correlates to be integrated with viral biomarkers, liver-disease severity, and close post-withdrawal monitoring rather than as stand-alone criteria for stopping therapy.

## 1 Introduction

Chronic hepatitis B (CHB), caused by hepatitis B virus (HBV), remains a major global health problem, affecting more than 250 million people and causing substantial cirrhosis- and hepatocellular-carcinoma-related mortality (1). Potent nucleos(t)ide analogue (NA) therapy suppresses HBV DNA replication and reduces liver-related risk, but it rarely eliminates the covalently closed circular DNA reservoir or integrated HBV DNA-derived antigen production. Confirmed hepatitis B surface antigen (HBsAg) loss, with undetectable HBV DNA and with or without antibody to HBsAg (anti-HBs), is the clinically relevant definition of functional cure; anti-HBs is not required for that definition (2–4).

Because HBsAg loss during continued NA therapy is uncommon, finite-treatment strategies have been investigated in carefully selected, predominantly non-cirrhotic hepatitis B e antigen (HBeAg)-negative patients. NA withdrawal can increase the chance of HBsAg loss or sustained off-treatment control, but it also produces frequent virological relapse and clinically important hepatic flares. Large cohorts and meta-analyses show that the magnitude of benefit and harm varies substantially across populations, treatment histories, NA types, and definitions of relapse or flare (5–8). The RETRACT-B program, for example, illustrates that a large proportion of patients experience post-withdrawal alanine aminotransferase (ALT) flares, whereas flare occurrence alone does not guarantee HBsAg loss (7).

Randomized and prospective studies frame the size of the clinical opportunity. In STOP-NUC, HBsAg loss occurred in 10.1% of participants assigned to stop therapy and in none assigned to continue, with the benefit concentrated among patients with end-of-treatment HBsAg below 1,000 IU/mL (9). In the global RETRACT-B cohort, the 48-month probabilities of sustained remission and HBsAg loss were 29.7% and 9.9%, respectively, whereas virological relapse and retreatment remained common (10). A 2025 meta-analysis of 62 studies and 9,867 participants estimated a pooled HBsAg seroclearance rate of 10%, but with very high heterogeneity and a strong dependence on follow-up duration and baseline population (11). Conversely, the Hepatitis B Research Network (HBRN) withdrawal study reported only 4.3% HBsAg loss by two years, and ALT flares were associated with subsequent immune-active disease rather than HBsAg loss (12). These divergent results make clear that withdrawal is not a uniform immune intervention; its outcome depends on the host-virus state in which antigen rebound occurs.

The clinical challenge is therefore not simply whether immune activation occurs after NA withdrawal, but whether the response is sufficiently organized to control HBV without causing disproportionate liver injury. Quantitative HBsAg, hepatitis B core-related antigen (HBcrAg), and serum HBV ribonucleic acid (RNA) provide information about residual viral transcription and relapse risk, but they do not fully capture the functional competence, specificity, or tissue localization of the host immune response (13–19). Immune correlates may complement these viral markers, although no immune assay has yet been validated as a stand-alone criterion for treatment discontinuation.

This Mini Review focuses on longitudinal human studies that directly examined immune parameters around NA withdrawal. Evidence from treatment-naive CHB, pegylated interferon-induced functional cure, liver single-cell studies, and animal models is discussed as mechanistic context rather than equivalent clinical evidence. The central framework is intentionally cautious: currently available immune signatures are candidate correlates of outcome, not proven causal determinants. **Table 1** summarizes the evidence hierarchy and distinguishes direct longitudinal withdrawal studies from adjacent or mechanistic evidence.

**Table 1:** Evidence hierarchy for immune correlates after NA withdrawal.

Evidence layer	Representative study	Design and sample	Main signal and principal limitation
Direct, longitudinal human evidence	Lens et al. (22)	21 HBeAg-negative patients; flow cytometry, bait staining, enzyme-linked immunospot assay; end of treatment, week 12, week 48	HBsAg-specific memory B-cell PD-1 reduction, plasmablast expansion, and stronger T follicular helper-cell association in HBsAg loss. Small cohort; observational.
Direct, longitudinal human evidence	Holmberg et al. (29); Hume et al. (23)	Nuc-Stop: 127-patient cohort with a 32-patient proteomic subset; HBsAg-anti-HBs immune-complex nested case-control, n=38	Soluble signatures, alanine aminotransferase variability, and HBsAg-anti-HBs immune-complex kinetics distinguish flare trajectories. Composite endpoints and limited external validation.
Direct/adjacent human evidence	Rivino et al. (24); Garcia-Lopez et al. (8)	Prospective NA-withdrawal cohorts with peripheral HBV-specific T-cell and viral measurements	Functional HBV-specific T cells at suppression associate with off-treatment control. Assays and sampling schedules differ between cohorts.
Direct systemic biomarker evidence	Liao et al. (32); d’Almeida et al. (45)	Prospective withdrawal cohorts evaluating end of treatment soluble PD-1 or combined HBV biomarkers	Feasible circulating markers may stratify HBsAg loss or severe flare risk. Biological specificity and external calibration remain limited.
Indirect mechanistic context	Le Bert et al. (25); Aliabadi et al. (26); Andreata et al. (34); Heim et al. (35); Bosch et al. (36)	Treatment-naive CHB, liver single-cell studies, or mechanistic human/mouse studies	Explain antigen burden, tissue tolerance, and co-signaling, but do not by themselves validate NA-withdrawal biomarkers.

## 2 Direct immune correlates in NA-withdrawal cohorts

### 2.1 B-cell competence and humoral recovery

HBsAg-specific memory B cells are detectable in CHB but are commonly atypical, express high levels of programmed cell death protein 1 (PD-1), and are poorly able to differentiate into antibody-secreting cells. Foundational studies showed that this defect can be partially relieved *in vitro* by PD-1 pathway inhibition together with cluster of differentiation 40 ligand and B-cell maturation signals, supporting biological reversibility but not yet establishing a clinical intervention (20, 21).

The most direct evidence for NA withdrawal comes from the prospective study by Lens and colleagues, which followed 21 HBeAg-negative patients after at least three years of virological suppression (22). During treatment, the frequencies of class-switched hepatitis B core antigen-specific and HBsAg-specific memory B cells became more similar, but the HBsAg-specific cells retained high PD-1 expression and remained functionally impaired. After NA withdrawal, patients who achieved HBsAg loss showed more activated global memory B cells, plasmablast expansion, reduced PD-1 expression on HBsAg-specific memory B cells, trends toward improved activation and antibody-secreting function, and a stronger relationship between these cells and circulating T follicular helper cells. The persistence of these changes years after HBsAg seroconversion is compatible with durable immune remodeling, but the small sample and observational design prevent causal inference.

The same study also illustrates why humoral responses should not be reduced to anti-HBs concentration alone. Increases in class-switched hepatitis B core antigen-specific memory B cells were temporally linked to hepatic flares, whereas the HBsAg-specific memory B-cell pattern was associated with HBsAg loss. These observations suggest that antigen specificity and cellular function may be more informative than total B-cell frequency. They also argue against describing anti-HBs production as the sole or final effector mechanism of functional cure, because HBsAg loss can occur without measurable anti-HBs and may reflect coordinated changes in viral transcription, infected-cell control, and antigen clearance (2).

The distinction between hepatitis B core antigen (HBc)- and HBsAg-directed responses may also help separate immune exposure from clinically useful humoral control. Core antigen is produced during renewed viral replication and can stimulate readily detectable HBc-specific responses, but these responses do not directly neutralize circulating virions or remove HBsAg. By contrast, HBsAg-specific B cells must recognize an antigen that circulates in large excess as non-infectious subviral particles and may be chronically driven toward atypical or exhausted phenotypes. A rise in hepatitis B core antigen-specific memory B-cell frequency during flare may therefore document antigen re-exposure, whereas recovery of HBsAg-specific memory B-cell differentiation and antibody-secreting capacity may be more closely related to antigen decline. This interpretation remains provisional because cellular assays, serum antibody measurements, and immune-complex assays capture different parts of the same process.

Future withdrawal studies should characterize the quality as well as the quantity of the humoral response. Relevant features include memory-B-cell phenotype, B-cell receptor clonality and somatic hypermutation, neutralizing activity, antibody Fc-mediated effector functions, plasmablast kinetics, and the formation and clearance of HBsAg-anti-HBs immune complexes. Total anti-HBs may remain negative when antibody is bound by excess HBsAg, making immune complexes potentially informative before conventional seroconversion becomes detectable (23). At the same time, high immune-complex levels could theoretically contribute to inflammation, so directionality should not be assumed. Paired antigen, free antibody, immune-complex, and cellular measurements are needed to determine whether the humoral signature is a driver of clearance, a consequence of falling antigen, or both.

### 2.2 T-cell fitness, inhibitory receptors, and timing of measurement

HBV-specific cluster of differentiation 8-positive (CD8^+^) T cells are central to viral control, but their function in CHB is heterogeneous and shaped by antigen exposure, age, treatment duration, and the liver microenvironment. In the seminal NA-withdrawal cohort of Rivino and colleagues, the presence of functional HBV-specific T cells during viral suppression was associated with subsequent off-treatment viral control, suggesting that immune competence at the point of withdrawal may matter more than a late, nonspecific expansion after viral rebound (24).

Studies outside the withdrawal setting provide useful but indirect context. In treatment-naive patients, age and duration of infection were stronger correlates of HBV-specific T-cell responsiveness than contemporaneous HBsAg level, while lower HBcrAg was associated with greater reversibility in response to programmed death-ligand 1 blockade (25, 26). A separate model linked later treatment initiation and longer treatment duration to the probability of detecting functional HBV-specific CD8^+^ responses and subsequent off-treatment control (27). These findings support a role for cumulative antigen exposure, but they should not be treated as validated NA-withdrawal selection rules.

The Hepatitis B Research Network provides an important caution about timing. In 34 adults treated with tenofovir for 192 weeks, HBV-specific T-cell function did not improve despite viral suppression. Post-treatment ALT flares were associated with higher percentages of PD-1-positive CD8^+^ T cells, PD-1-positive cluster of differentiation 4-positive T cells, and cytotoxic T-lymphocyte-associated protein 4-positive cluster of differentiation 4-positive T cells measured at the pre-treatment baseline, but flares were not accompanied by HBsAg loss or improved HBV-specific T-cell function (28). These data support the idea that global inhibitory-receptor expression may mark a propensity to inflammatory flares, but they do not prove that the same phenotype measured at end of treatment predicts outcome.

The apparent dual role of PD-1 requires careful population definition. PD-1 on antigen-specific cells can indicate ongoing antigen recognition and functional restraint, whereas PD-1 on broad peripheral T-cell populations may reflect an exhaustion-prone or inflammatory state. Future studies should report antigen specificity, co-expression of multiple inhibitory receptors, differentiation state, and functional readouts rather than treating PD-1 as a unidirectional marker.

### 2.3 Soluble mediators, ALT dynamics, and immune complexes

The prospective Nuc-Stop study provides the most detailed soluble immune analysis currently available in NA withdrawal. Among 127 HBeAg-negative participants followed for 36 months, flares were common under the study’s definition of ALT greater than twice the upper limit of normal or twice baseline. A proteomic analysis in a subset of 32 participants without treatment restart distinguished good flares, defined by HBsAg loss, a greater than 1 log10 HBsAg decline, or sustained viral control, from bad flares lacking both HBsAg decline and virological control (29).

At the end of treatment, good flares were associated with higher interleukin 13, tumor necrosis factor-related apoptosis-inducing ligand, and receptor activator of nuclear factor kappa-B ligand, whereas bad flares showed higher C-X-C motif chemokine ligand 11, osteoprotegerin, tumor necrosis factor, and, at some time points, interleukin 7. After the initial ALT spike, good flares also displayed lower ALT variability than bad flares. These findings are biologically plausible but exploratory. The proteomic sample was small, the flare outcome was composite, and the panel was a targeted multiplex assay rather than an unbiased discovery platform. The markers should therefore be described as hypothesis-generating signatures requiring external validation.

An earlier analysis of the same cohort linked clinical relapse after cessation to increases in C-X-C motif chemokine ligand 10, tumor necrosis factor, interleukin 9, interferon gamma (IFN-gamma), macrophage inflammatory protein 1 beta, and interleukin 12 (30). Because both analyses arise from the same underlying cohort, they should not be treated as independent validation studies. Replication across cohorts with prespecified endpoints and correction for multiple testing is needed.

HBsAg-anti-HBs immune complexes offer a complementary humoral readout. In a nested case-control analysis of 38 non-cirrhotic HBeAg-negative patients, HBsAg-anti-HBs immune complexes were detectable in 97% at end of treatment. High end-of-treatment levels were less frequent among participants who developed a hepatitis flare, and peak off-treatment immune-complex levels were higher in participants with a substantial HBsAg decline (23). The study is important because it measures antibody that may be masked by circulating antigen, but its sample size and case-control design limit generalizability. The observed lower HBsAg-anti-HBs immune-complex levels among tenofovir-treated participants may contribute to differences between NA regimens, but this remains a hypothesis rather than a demonstrated mechanism.

### 2.4 Time-resolved interpretation of immune activation

Immune measurements after NA withdrawal should be interpreted as trajectories rather than isolated values. Virological rebound usually precedes biochemical activity, creating at least three biologically distinct windows: the end-of-treatment state, early antigen re-exposure, and the subsequent inflammatory or control phase. In a prospective multicentre cohort of 110 non-cirrhotic HBeAg-negative patients, virological reactivation occurred in 109 participants and was detected earlier after tenofovir disoproxil fumarate than after entecavir withdrawal; nevertheless, ALT flares were not associated with HBsAg loss (31). Thus, an immune marker obtained at the end of treatment may reflect pre-existing competence, whereas the same marker measured during rebound may reflect activation, redistribution, tissue recruitment, or injury.

Soluble PD-1 illustrates both the promise and the interpretive difficulty of longitudinal biomarkers. In 115 non-cirrhotic patients, detectable end-of-treatment soluble PD-1 was associated with a lower cumulative incidence of clinical relapse and a higher probability of HBsAg loss over long-term follow-up (32). This finding is clinically interesting because the assay is simpler than antigen-specific cellular testing. However, soluble PD-1 cannot be assumed to represent membrane PD-1 on HBV-specific T cells, and its cellular source, biological activity, and optimal cut-off require external validation. It should therefore be considered a candidate systemic correlate, not interchangeable with flow-cytometric PD-1 expression.

The flare itself is also temporally heterogeneous. An early, self-limited ALT increase accompanied by falling HBsAg and subsequent viral control differs from recurrent ALT oscillation with persistently high HBV DNA and no antigen decline. Small proof-of-concept observations have linked marked HBsAg decline during severe flares to strong IFN-gamma and tumor necrosis factor activity, but these data include only a few patients and cannot establish that delaying retreatment is safe (33). The clinically useful signal may therefore lie in the joint slope and timing of HBV DNA, HBsAg, ALT, and immune mediators rather than in the peak value of any one variable.

## 3 Mechanistic context beyond direct withdrawal studies

### 3.1 Liver-intrinsic constraints on T-cell function

Peripheral blood is an imperfect window into the liver. Recent single-cell and spatial studies suggest that intrahepatically primed HBV-specific CD8^+^ T cells may enter a dysfunctional state early and may be relatively resistant to PD-1 blockade alone. Co-stimulatory pathways such as tumor necrosis factor receptor superfamily members 9 or 4, attenuated-effector cell states restrained by transforming growth factor beta, and a liver sinusoidal endothelial cell adenylyl cyclase/cyclic adenosine monophosphate/protein kinase A rheostat provide complementary explanations for why peripheral inhibitory-receptor measurements do not consistently predict intrahepatic antiviral activity (34–36).

These findings are mechanistically relevant to NA withdrawal because viral rebound occurs in a tissue with tolerogenic endothelial, myeloid, and stromal networks. Nevertheless, none of these studies directly tested a standardized biomarker panel before NA cessation. They should therefore be used to generate hypotheses about tissue-specific markers, not to imply that a circulating PD-1 measurement captures the whole liver response.

### 3.2 Innate and suppressive networks

Dendritic cells, myeloid-derived suppressor cells, regulatory T cells, natural killer cells, and unconventional T cells can shape the quality of adaptive immune activation. Chronic HBV and HBsAg exposure impair antigen presentation, sustain myeloid-derived suppressor-cell-regulatory T-cell circuits, and create a paradoxical natural-killer-cell state in which activating receptors and effector functions are discordant (37–42). Liver single-cell studies further identify macrophage, mast-cell, liver sinusoidal endothelial-cell and regulatory T-cell networks that can either support cross-priming or reinforce type 2 immunosuppression (43).

This section is best interpreted as a biological map of potential modifiers rather than as evidence that innate-cell assays are ready for patient selection. In particular, findings from pregnancy-associated CHB or treatment-naive clinical phases should not be extrapolated directly to non-cirrhotic adults stopping long-term NA therapy.

### 3.3 The role of antigen burden and humoral history

The interaction between antigen burden and immune competence is likely nonlinear. Lower HBsAg and HBcrAg may reduce inhibitory antigen exposure, but an intrinsically dysfunctional B- or T-cell compartment may remain unable to exploit that lower burden. Conversely, a high antigen load can coexist with measurable immune competence in selected patients. This argues for combined models that include viral markers, age and treatment history, cellular function, and a small number of soluble or humoral markers rather than a single threshold.

Evidence from pegylated interferon-induced functional cure supports the value of combining HBcrAg with anti-HBc and anti-HBs for recurrence risk stratification, but that model was developed after interferon-based functional cure and should not be presented as a validated NA-withdrawal model (44).

The prospective analysis by d’Almeida and colleagues moves this principle closer to the withdrawal setting. Among 85 participants completing 48 weeks of follow-up, severe virological relapse was associated with detectable HBcrAg, while severe biochemical flare was associated with non-A genotype, detectable HBV RNA, and lower anti-HBc immunoglobulin G. A composite score based on HBcrAg, HBV RNA, and anti-HBc immunoglobulin G achieved a high negative predictive value for severe flare (45). Importantly, this is a virological-humoral risk model rather than a complete measurement of immune competence. Its main contribution is to show that markers of residual viral activity and humoral exposure history can identify a safer substrate in which more specific immune markers might add value.

### 3.4 Spatial compartmentalization and non-cytolytic control

A further limitation of blood-based biomarker discovery is that successful control does not necessarily require wholesale cytolytic elimination of infected hepatocytes. HBV-specific CD8^+^ T cells can suppress replication through IFN-gamma- and tumor necrosis factor-mediated non-cytolytic mechanisms, while B cells and antibodies can reduce circulating antigen and limit viral spread. In contrast, indiscriminate cytotoxicity may raise ALT without producing durable antigen decline. The balance between non-cytolytic control, selective removal of infected cells, and bystander injury is therefore central to the distinction between a productive and a harmful flare (17, 34–36).

This balance is strongly conditioned by location. Sinusoidal endothelial cells, Kupffer cells, monocyte-derived macrophages, stellate cells, and tissue-resident lymphocytes shape antigen presentation and effector access within the liver. Peripheral expansion may be beneficial if it reflects recruitment of functional HBV-specific clones, but unhelpful if the cells remain excluded, metabolically constrained, or dominated by nonspecific inflammatory populations. Paired blood and liver sampling is rarely feasible in withdrawal trials, yet emerging single-cell, T-cell receptor, cell-free RNA, extracellular-vesicle, and proteomic approaches may provide partial tissue surrogates (19, 34–36, 43). These technologies should be benchmarked against clinically relevant outcomes before being incorporated into stopping algorithms.

## 4 Toward immune-informed NA discontinuation

### 4.1 A staged biomarker model

**Figure 1:**
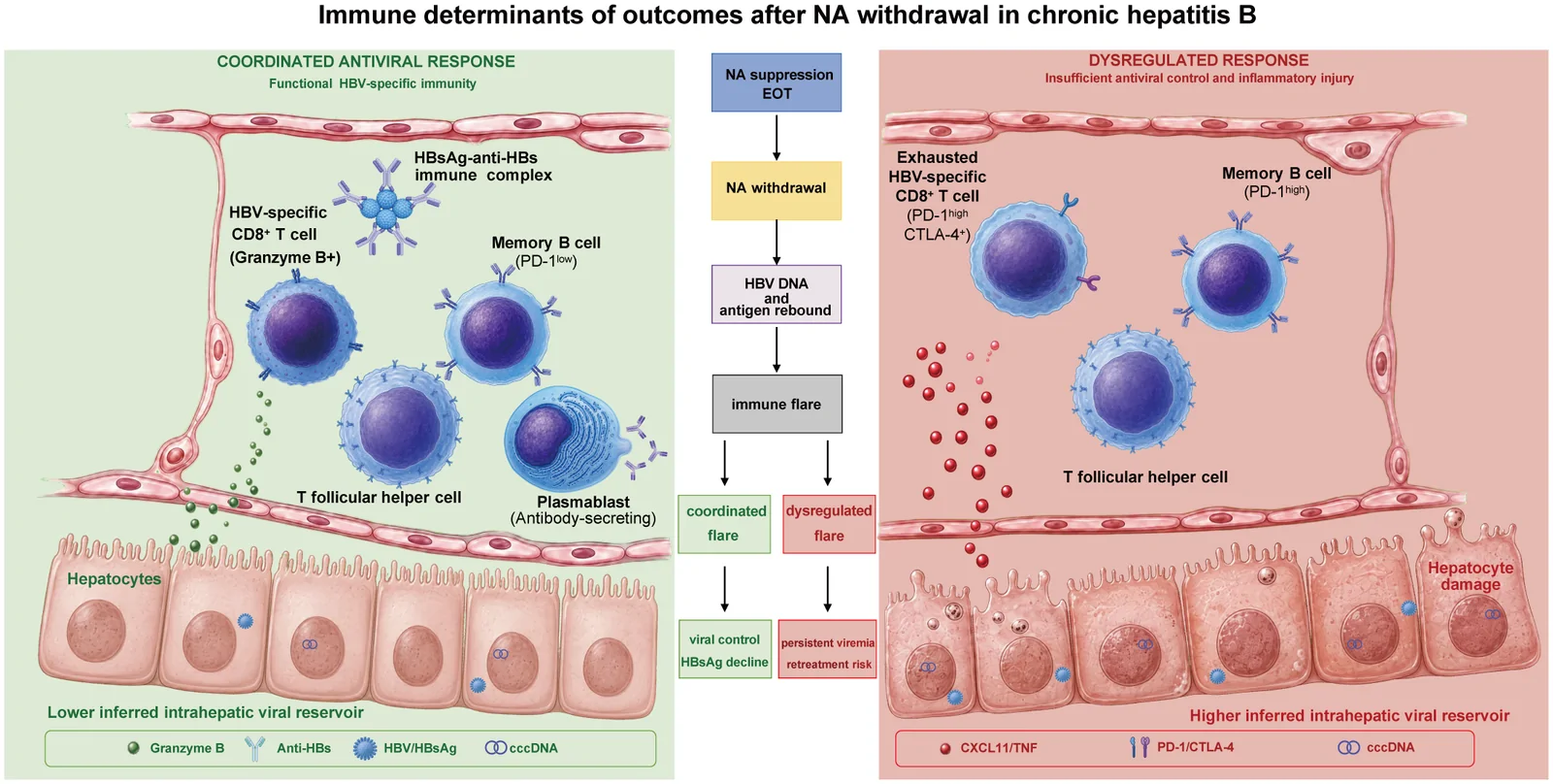
Proposed time-resolved framework for immune outcomes after nucleos(t)ide analogue withdrawal. The schematic illustrates how the immune state at the end of treatment may influence the response to hepatitis B virus and antigen rebound after treatment withdrawal. A coordinated antiviral response is characterized by preserved virus-specific CD8-positive T-cell activity, recovery of HBsAg-specific memory B-cell function, plasmablast expansion, T follicular helper-cell support, and the formation of HBsAg-anti-HBs immune complexes. These features may contribute to controlled inflammation, viral control, and HBsAg decline. In contrast, a dysregulated response is associated with exhausted virus-specific T cells, increased inhibitory-receptor expression, impaired memory B-cell function, inflammatory mediators, hepatocyte injury, persistent viremia, and an increased likelihood of retreatment. The inferred differences in the intrahepatic viral reservoir are conceptual and have not been directly validated as determinants of withdrawal outcomes. The illustrated immune markers are candidate correlates and should not be interpreted as validated stand-alone criteria for treatment discontinuation. anti-HBs, antibodies to hepatitis B surface antigen; cccDNA, covalently closed circular DNA; CD8, cluster of differentiation 8; CTLA-4, cytotoxic T-lymphocyte-associated protein 4; CXCL11, C-X-C motif chemokine ligand 11; DNA, deoxyribonucleic acid; EOT, end of treatment; HBsAg, hepatitis B surface antigen; HBV, hepatitis B virus; NA, nucleos(t)ide analogue; PD-1, programmed cell death protein 1; TNF, tumor necrosis factor.

The current evidence supports three cautious principles. First, B-cell competence is clinically relevant, but it should be assessed through antigen-specific phenotype and function where possible. A potential panel would include activation and PD-1 expression of HBsAg-specific memory B cells, plasmablast frequency, association with T follicular helper cells, and HBsAg-anti-HBs immune-complex levels, interpreted alongside HBcrAg and HBsAg (22, 23). Second, T-cell assessment should distinguish global inhibitory-receptor expression from antigen-specific function and should record whether measurements were obtained before treatment, at end of treatment, or after withdrawal. Third, soluble marker panels and ALT trajectories may help distinguish a controlled flare from prolonged inflammatory injury, but thresholds and composite endpoints require prospective validation (28–32). **Figure 1** summarizes this time-resolved distinction between a coordinated antiviral response and a dysregulated inflammatory trajectory.

A clinically usable algorithm will need to integrate four domains: (i) virological state, including HBsAg, HBcrAg and, where available, HBV RNA; (ii) immune competence, including B- and T-cell specificity and function; (iii) liver reserve and fibrosis stage; and (iv) an explicit monitoring and retreatment plan. Patients with cirrhosis, limited liver reserve, or inability to comply with intensive monitoring should not be considered appropriate candidates solely because an immune marker appears favorable (2–4). Immune biomarkers should refine risk assessment, not replace established safety criteria.

A staged design is more realistic than a single universal panel. Stage 1 should use widely available safety and virological variables to exclude unsuitable patients and estimate the probability of severe relapse (13–16, 45). Stage 2 could apply a compact immune panel only to the clinically eligible group, enriching for antigen-specific B-cell competence, functional HBV-specific T cells, or favorable soluble profiles (22–24, 29, 32). Stage 3 begins after withdrawal and uses dynamic HBV DNA, HBsAg, ALT, bilirubin, and selected immune measurements to classify the trajectory. This sequence limits expensive testing, separates prediction from monitoring, and prevents a favorable experimental immune result from overriding established clinical contraindications.

### 4.2 Flares, retreatment, and the safety boundary

The concept of a beneficial flare also requires refinement. A flare that produces a short-lived HBsAg decline, sustained viral control, or HBsAg loss may not have the same biological meaning, and a composite endpoint can obscure clinically important differences (7, 10, 12, 29–31). Future cohorts should prespecify separate outcomes for virological relapse, biochemical flare, severe flare, HBsAg decline, HBsAg loss, retreatment, hepatic decompensation, and death.

Clinical cohorts show why retreatment cannot be treated simply as failure or censoring. Early retreatment may prevent severe liver injury but can truncate the antigenic and inflammatory sequence being studied; delayed retreatment may preserve an opportunity for immune control but expose patients to avoidable harm. In HBRN, HBV DNA above 4 log10 IU/mL at the preceding visit identified increased flare risk, and flares were not associated with later HBsAg loss (12). RETRACT-B similarly found that profound or recurrent virological and biochemical relapses during the first year predicted less favorable long-term remission (10). These observations argue against tolerating repeated or escalating flares merely in the hope of functional cure.

Withdrawal studies should therefore distinguish protocol-mandated retreatment, clinician-discretion retreatment, and patient-preference retreatment. They should also report the biomarker values immediately before retreatment, because these samples define the boundary between a potentially productive immune response and an unacceptable safety trajectory. Models intended for practice must be evaluated for both missed severe flares and unnecessary retreatment, not only for HBsAg loss.

## 5 Limitations and research priorities

The evidence base has four major limitations, as reflected across available withdrawal cohorts and nested immune studies (5, 7, 10–12, 22, 23, 28, 29, 32, 45). First, the most detailed immune studies are small and often nested within larger cohorts, so apparent associations may be unstable and vulnerable to multiple-testing bias. Second, sampling time points vary widely, and the term “baseline” may refer to treatment initiation rather than end of treatment. Third, flare and beneficial-response definitions are not harmonized, which limits cross-study comparison. Fourth, most cohorts are non-cirrhotic, regionally concentrated, and insufficiently powered for analyses by HBV genotype, sex, ethnicity, NA type, or route and duration of infection.

Consistent with current biomarker-development recommendations, the next generation of studies should prespecify immune hypotheses, recruit multicentre and multiethnic populations, standardize flow-cytometry and immune-complex assays, and collect paired end-of-treatment, early post-withdrawal, flare, and recovery samples. External validation should be separated from model development, with calibration and clinically meaningful decision curves reported in addition to the area under the receiver-operating-characteristic curve (14, 19). Liver tissue or minimally invasive tissue surrogates will be important for testing whether peripheral markers reflect the compartments that actually control viral rebound.

Assay harmonization deserves particular attention. Flow-cytometry reports should specify peptide pools or bait reagents, stimulation duration, gating strategy, inhibitory-receptor combinations, and whether frequencies are expressed among total or antigen-specific lymphocytes. Functional assays should measure more than IFN-gamma alone and should ideally include proliferation, polyfunctionality, cytotoxic potential, and rescue after checkpoint or co-stimulatory perturbation. Soluble-marker studies should report pre-analytic handling, lower limits of detection, batch correction, missingness, and multiplicity control. Candidate thresholds should be locked before external testing rather than optimized repeatedly within small discovery cohorts (22, 23, 28–32).

Mechanistic intervention studies are also needed. If a signature identifies an immune deficit rather than a protective state, the appropriate response may be antigen reduction, therapeutic vaccination, transforming growth factor beta modulation, co-stimulatory agonism, or another combination strategy rather than NA withdrawal alone. The adeno-associated virus-HBV model may provide a tractable platform for testing liver-specific immune events (46), whereas hepatitis C virus cure studies suggest that residual immune dysfunction can persist after antigen clearance (47). Studies of checkpoint blockade, tumor necrosis factor receptor superfamily member 9/4 signaling, and small interfering RNA or antisense-mediated antigen reduction should therefore incorporate standardized immune endpoints so that biomarkers can be tested as predictors of mechanism, safety, and durability (48).

The contrast between withdrawal alone and immune-active transition strategies is informative. Switching selected HBeAg-negative patients from NA therapy to 48 weeks of pegylated interferon reduced relapse and increased HBsAg loss compared with cessation alone in a randomized trial (49), whereas an HBRN trial of prolonged tenofovir with or without an initial short course of pegylated interferon showed earlier but not higher overall HBsAg clearance after protocolized withdrawal (50). These studies do not validate interferon for every candidate, but they support a broader principle: withdrawal outcomes may improve when antigen reduction and immune stimulation are deliberately sequenced rather than relying on uncontrolled rebound as the only immune trigger.

## 6 Conclusion

NA withdrawal exposes a clinically important interaction between viral rebound and a pre-existing immune landscape. The strongest direct evidence currently points to three candidate correlates of outcome: recovery of HBsAg-specific B-cell competence, preserved or appropriately targeted HBV-specific T-cell function, and a soluble/ALT trajectory compatible with controlled rather than persistent inflammation. HBsAg-anti-HBs immune complexes may add a functional humoral dimension. However, these markers are not yet validated predictors and should not be used in isolation to decide whether treatment can be safely stopped. The field now needs harmonized definitions, standardized assays, external validation, and prospective models that combine viral, immune, and liver-safety information.
